# Institutionalization of integrated community case management into national health systems in low- and middle-income countries: a scoping review of the literature

**DOI:** 10.1080/16549716.2019.1678283

**Published:** 2019-11-07

**Authors:** Agnes Nanyonjo, Helen Counihan, Sam Gudoi Siduda, Kassahun Belay, Gloria Sebikaari, James Tibenderana

**Affiliations:** a Technical Department, Malaria Consortium Uganda, Kampala, Uganda; b Technical Department, Malaria Consortium, London, UK; c Management Department, USAID’s Malaria Action Program for Districts, Kampala, Uganda; d Technical Department, US President’s Malaria Initiative, US Agency for International Development, Kampala, Uganda

**Keywords:** Institutionalization, iCCM, integration, national health systems

## Abstract

**Background**: Integrated community case management (iCCM) for malaria, pneumonia and diarrhea continues to be a recommended strategy to address child mortality in areas where access to health facilities is limited.

**Objective**: To identify models of, and gaps in, institutionalization of benchmark components of iCCM into national health systems of low-and-middle-income countries, in order to draw lessons for future iCCM implementation and sustainability.

**Methods**: A scoping review of relevant searchable policy documents and publications available in English literature was undertaken. Data were selected, collated and characterized by three reviewers using the Arksey and O’Malley framework.

**Results**: Overall 19 countries were reviewed. Despite the existence of discrete policies, most iCCM programs relied heavily on implementing partners and donor financing. Parallel implementing partner-run systems were often used to procure and supply iCCM medicines. These modes of implementation occasionally violated some health system strengthening principles. Drug stock-outs were still prominent in several countries, and iCCM indicators were sometimes not integrated into the national health management information system. There were no clearly defined motivation packages for both salaried and unsalaried workers, and there were several supervision challenges. Community-based performance-financing, use of technology with mobile devices (mHealth), small procedural improvements, and provision of targeted rather than universal services, were some of the promising interventions for improved iCCM institutionalization.

**Conclusion**: Sustainable iCCM will require improved ownership by the benefiting communities and the local and central governments. Government commitment should be evident in budgeting processes and implementation strategies.

## Background

Globally, child mortality has significantly declined over the past few decades [1]. Nevertheless, inequalities continue to be observed between and within countries [2]. Malaria, pneumonia and diarrhea disproportionately affect children in low- and middle-income countries, especially those from poor households with limited geographical and financial access to quality services provided by formal health facilities [1]. Combined, they account for approximately 40% of mortality among children aged five and below [3,4]. The World Health Organization (WHO) and United Nations Children’s Fund (UNICEF) recommend integrated community case management (iCCM) as a key child survival strategy [3,5]. iCCM is an equity-based strategy to equip, train, support and supervise community health workers (CHWs) to deliver to these children life-saving treatment interventions for malaria, pneumonia and diarrhea [6]. If implemented under optimal conditions (including presence of equipment and uninterrupted commodities for well trained and supported CHWs), iCCM is expected to be an effective method of delivering prompt appropriate treatment for all three conditions in high mortality countries [3,5].

Therefore, iCCM should be seen as an integral part of the health system response involving health promotion, disease prevention, and treatment for common childhood conditions in resource-limited settings rather than an independently managed program [7]. However, successful transition from independently managed and typically externally funded iCCM programs to more integrated programs requires the existence of adequate policies addressing benchmark components of iCCM. These components include coordination and policy setting; costing and financing; human resources; supply chain management; service delivery and referral; communications and social mobilization; supervision and performance quality assurance; and monitoring, evaluation and integration into health information systems [5,8]. Furthermore, the scalability of iCCM programs requires their incorporation into national level priorities with corresponding funding and sustainability capabilities [7]. All these aspects align with principles of health system strengthening, both in general and more specifically for community-based primary healthcare [9,10]. Yet, in many countries with high child mortality, donor support has tended to focus on vertical programs reliant on external funding to the detriment of more government-led approaches [11].

Although a few countries have partially or fully integrated iCCM into their national health systems [12], there is limited research synthesis mapping out individual models of the institutionalization of community-based health strategies including iCCM [13]. This article draws on scoping review methodology to give an overview of approaches that have been used to institutionalize iCCM into national health systems; characterize their successes and limitations; and discuss their implications for policy and program sustainability.

## Methods

### Scoping review protocol

The scoping review methodology is recommended for studying complex and non-extensively reviewed topics [14]. The study used this methodology, whereby the state of knowledge about an issue is documented to identify knowledge gaps, set research agendas and identify implications for decision-making [15]. Specifically, it aimed to map existing literature on the institutionalization of iCCM into national health systems in order to identify models, gaps and lessons.

The study was based on the six-stage scoping review protocol developed by Arksey and O’Malley, modified by Levac et al., and further refined by Peters et al. [15–17]. Hence, a research question – ‘what approaches have been used to institutionalise iCCM into government led and national health systems?’ – was determined, relevant studies identified, studies selected, data charted, and the results collated, summarized and reported. The optional sixth step, stakeholder consultation, was omitted in this review as results from the exercise are reported in a separate report.

### Identifying the relevant literature

A search strategy was developed to identify relevant national and international iCCM policy and implementation resource documents, online published research articles, and grey literature reports from low-income African countries and elsewhere. The strategy involved: i) a systematic search of relevant reference databases and websites including country-specific Ministry of Health (MoH) websites, key funding agencies’ websites, PubMed, ccmcentral.com and chwcentral.com; and ii) identification of relevant referenced sources. The databases and websites were searched using a combination and truncation of key-words including ‘integrated’, ‘community’, ‘case management’, ‘community health worker’, ‘community-based’, ‘management of childhood illnesses’, ‘malaria’, ‘pneumonia’ and ‘diarrhea’.

Chronologically, the first step in the systematic search involved application of the search criteria to PubMed to identify countries that had published about iCCM since 2012. This cut-off date was linked to the issuing of the joint statement on iCCM by WHO/UNICEF in 2012 [3]. The second step involved sourcing for grey literature and relevant policy documents from the additional-aforementioned sources. This was followed by a hand search for relevant referenced sources. However, key or sole iCCM implementation guiding documents written before 2012 were exempted from the 2012 cut-off point.

### Citation management

All citations were imported into Endnote X7.3.1 (Thomson Reuters), and duplicates removed in preparation for title and abstract screening.

### Inclusion criteria

Firstly, any included document had to be from a low or middle-income country that was implementing iCCM beyond its early stages and which had sufficient literature on iCCM in the English language that provided discrete pieces of information related to the research question. Pilot and feasibility studies implemented in small areas by independent organizations with tightly controlled protocols, outside national health systems and government policy, were excluded from the review because these usually do not experience the difficulties and practicalities encountered in large and nationwide implementation of iCCM. Secondly, any included document, report or research article had to have iCCM (as described by the WHO/UNICEF joint statement) in a low or middle-income country as its main focus. Finally, only reports and research articles with elements of government-led institutionalization of any of the benchmark components of iCCM at scale were retained.

### Methodological quality appraisal

Methodological quality was not appraised as this was not prescribed by the scoping review methodology [15]. Furthermore, the institutionalization of integrated programs like iCCM is complex and has been studied using several non-experimental designs (both quantitatively and qualitatively), the strengths of which cannot be evaluated using available guidelines.

### Selecting the relevant literature

The initial review team (AN, JT and HC) screened titles and abstracts of published journal articles for relevance, based on the aforementioned criteria. Those that fulfilled the criteria were read in detail for subsequent data characterization. The most recent government resources or policy documents that comprehensively referred to iCCM were all included in the review for data characterization.

### Data collation and characterization

In order to extract data, a spreadsheet was created in Microsoft Excel 2010 (Microsoft Corporation, Redmond, WA). Therein, the authors captured documents’ country of origin, year of publication, citation type, study type (where applicable), funders, implementing partners, and general model of iCCM implementation described. Also recorded were descriptions pertaining to the broad research question with a focus on benchmark components of iCCM that need to be institutionalized into the national health system. The details were extracted by reading full-text articles and charting findings into the spreadsheet. These were recorded independently by the core team (AN, JT and HC) and discussed with the wider team of authors (SG, GS, and KB).

### Data synthesis and reporting

The data abstracted through the review exercise were summarized and thematically analyzed according to benchmark components of iCCM by the core team. The analysis was deductive: journal articles’ results and conclusions sections and whole texts of grey literature were explored for concepts and key limitations, implications of the various institutionalization models and approaches, and their implications for policy and practice are reported.

## Results

### Literature search and selection results

**Figure 1.**
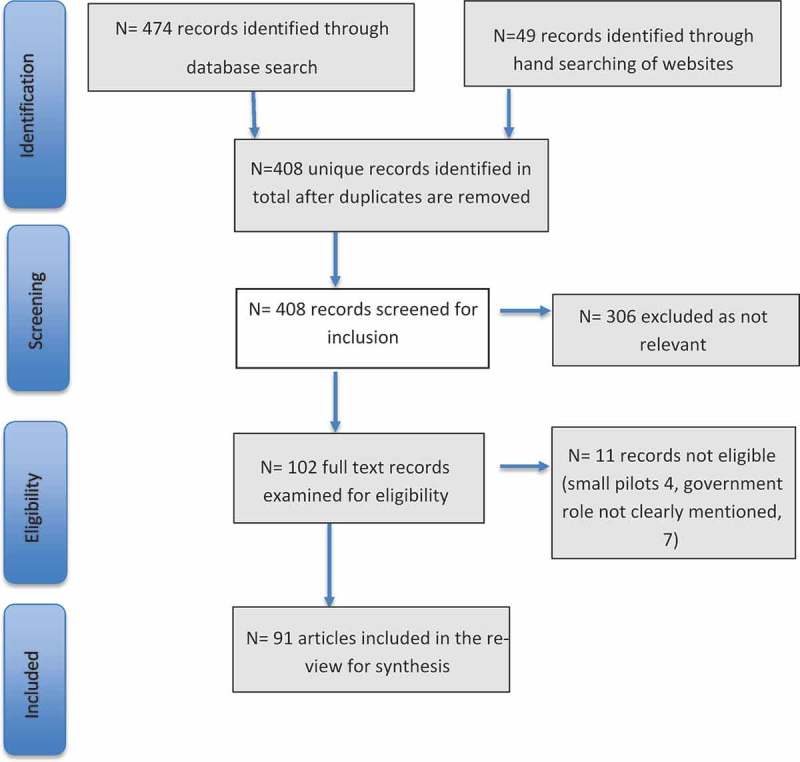
Flow chart of the search strategy results.

Conducted in August 2018, the search returned 474 potentially relevant records, including 49 records identified through hand searching of relevant institutional websites for Ministries of Health, international and non-governmental organizations, and key donors. After assessment of abstracts and titles for relevance, 128 full-text records were screened for eligibility. Government documents on iCCM were mainly national iCCM policies, implementation and training guidelines, CHW strategies and health sector strategic plans. Articles and documents from Angola, Cambodia, Guinea, Kenya, Liberia, Myanmar, Sierra Leone, The Gambia, and Togo were eliminated from the review pool due to either: i) being in very early stages of iCCM implementation so that no institutionalization messages could be captured, or ii) having insufficient literature online in the English language to enable evidence synthesis. After screening for eligibility and relevance, a total of 91 records were included in the review. Figure 1 summarizes the review process from identification to final inclusion. The full list of records included in the review is available as a supplementary file.

### General characteristics of citations

A total of 91 records were analyzed from 19 countries (16 African, one Central American and two Asian). The majority of these were from eastern (48%) and western (32%) Africa; journal articles (43%) with mainly quantitative study designs (Table 1); and written between 2015 and 2017 (45%). Although there were many publications about iCCM, only 8% of the readily searchable documents were national iCCM policy documents or strategies.

**Table 1.** Characteristics of included citations.

Characteristics of included records	Count N (%)
**Year of publication**	**N = 91**
2010–2011	3 (3)
2012–2014	32 (35)
2015–2017	41 (45)
2018	15 (16)
**Publication type**	**(N = 91)**
Journal article	39 (43)
Government document	7 (8)
Implementing partner report/study	32 (35)
Donor report	10 (11)
Meeting proceeding/conference proceeding	2 (2)
International guideline	1 (1)
Percentage representation of WHO Region of origin in literature*	(N = 88)*
East Africa	44 (48)
Central Africa	7 (8)
West Africa	29 (32)
South-central Asia	7 (8)
Central America	1 (1)
**Summary of studies by study design (where applicable)**	**N = 50**
Empirical qualitative	9(18)
Empirical quantitative	15 (30)
Empirical mixed and multiple method study designs	9 (18)
Literature reviews/synthesis	7 (14)
Case studies	5 (10)
Commentaries/Editorials/view points	5 (10)

* Values do not add to 100 due to double counting for citations that cover more than one region

### Policy setting and governance of iCCM

Governments of countries implementing iCCM had several development partners who propelled the iCCM agenda through existing community health system structures [8,9,18–21]. Subsequently, the management and coordination of iCCM were often shared between relevant MoH departments and their key implementing partners through core or technical working groups. The Ministries of Health were generally responsible for formulating policies and setting standards of management of malaria, pneumonia, and diarrhea by CHWs, as well as any other community packages provided alongside iCCM. The governments of countries with large-scale iCCM programs (e.g. Ethiopia, Ghana, Rwanda and Malawi) tended to strongly endorse global development policies and robust government ownership of iCCM programs as reflected in their country-specific iCCM strategies [8,20,22–24]. Some countries had separate iCCM policies or guidelines (e.g. Niger and Nigeria), while others had iCCM policies that were spread across several documents (e.g. Mali). However, iCCM policies were often embedded in national child health strategies (e.g. Burkina Faso, Ethiopia, Malawi, Mali, Nicaragua, Uganda, and Zambia), integrated community-based strategies (e.g. Cameroon, Madagascar, Rwanda and Senegal), and/or national malaria strategies (e.g. Ghana). In some countries, CHWs are authorized to deliver iCCM albeit without a comprehensive iCCM document or documents that were still being developed (e.g. the Democratic Republic of the Congo). Although on paper, consortia of implementing partners were often required to use a harmonized approach under the stewardship of the government, this did not always materialize in practice.

### Costing and financing

Most countries did not have long-term plans for financing iCCM, often depending on donor funding and technical support from implementation partners [21]. The implications of such models of funding for future sustainability and budgets were often raised even from within individual government institutions [8,25]. Despite their importance, recurrent costs for training, procurement of iCCM drugs, and maintenance of supply chain systems were usually excluded from budgetary forecasts. In some countries, such costs were instead covered by different donors and implementing partners [19,26–28]. In order to avoid reliance on external funding, some donor schemes (e.g. USAID and Global Fund) sometimes required governments to take on the responsibility of paying salaried and incentivized CHWs [29]. A few countries had already developed costed iCCM plans with technical support from their implementing partners and a couple more were in the process of doing so (Cameroon, Democratic Republic of Congo (DRC), Ethiopia, Ghana, Malawi, Nigeria, Rwanda, Senegal, South Sudan and Zambia). The costed plans outline the investments that were needed by government to scale iCCM to particular levels of implementation [19,26–28]. This was necessary because of the recurrent costs in training, supervision, stipends and salaries, regular supplies of iCCM drugs and supply chain systems to deliver the drugs [26].

### Human resources

The profiles of CHWs varied from country to country. CHWs were either community volunteers (e.g. Cameroon, DRC, Madagascar, Rwanda, Uganda and Nepal) or salaried workers (e.g. Ethiopia, Niger, Malawi and Pakistan). They were selected by the communities served, underwent training according to a well-defined curriculum and receive supervision from health facility staff or a higher cadre of CHWs. Often, the selection criteria required CHWs to belong to a particular age bracket, gender and level of literacy. For example, Burkina Faso, Ghana, Rwanda and Uganda specifically required that for each community, there is at least one male and one female CHW providing iCCM services. Conversely, Ethiopia’s Health Extension Workers (HEWs), Nepal’s Female Community Health Volunteers (FCHV) and Pakistan’s Lady Health Workers are all iCCM programs that rely solely on female community health workers for service delivery. Table 2 summarizes selected characteristics of CHWs implementing iCCM in various countries.

**Table 2.** Summary of characterises of iCCM community health workers.

Payment	Receive salary or stipends	No payment
	Burkina Faso, Ethiopia, Ghana-CBAs, Madagascar, Malawi, Mali, Mozambique, Niger, Uganda-CHEWs, Zambia-CHAs, Pakistan	Cameroon, DRC, Ghana-CHOs, Nigeria, Rwanda, Senegal, South Sudan, Uganda-VHTs, Zambia-others CHWs, Nicaragua, Nepal
**Incentives and motivation**	**Monetary**	**Non monetary**
	Burkina Faso before 2014, Madagascar (drug sellers)	Cameroon, Mali, Nigeria, Senegal, South Sudan, Uganda (implementing partners and community tokens)
	Rwanda, Madagascar (performance based financing)	Malawi (support from village committee)
	Access to loans (Nepal)	All (event based motivation)
**iCCM training**	**short iCCM training (5–14 days)**	**iCCM part of longer training**
	Burkina Faso, Cameroon, DRC, Ghana-CBAs, Madagascar, Malawi, Mozambique, Niger, Nigeria, Rwanda, Senegal, South Sudan, Uganda	Ethiopia, Ghana-CHOs, Zambia, Nicaragua, Pakistan
**Basic CHW training**	**Short courses (5–14 days)**	**longer courses (1–10 months)**
	Madagascar, Senegal, Uganda, Nicaragua, Nepal	Ethiopia, Ghana-CHOs, Zambia, Malawi, Mali, Mozambique, Niger, Pakistan
**Gender**	**One male and one female per community**	**No requirement**
Gender balance required	Burkina Faso, Ghana, Rwanda, Uganda	Cameroon, DRC, Madagascar, Malawi, Mali, Mozambique, Niger
Gender restricted to only females	Ethiopia, Nepal, Pakistan	
**Location of work**	**Home**	**Health post, health huts and village health compound or clinics**
	Burkina Faso, Cameroon, DRC, Pakistan, Nepal, Nicaragua, Ghana-CBAs, Madagascar, Mozambique, Nigeria, Rwanda, South Sudan, Uganda-VHTs, Zambia-other CHWs	Ethiopia, Ghana-CHOs, Malawi, Mali, Niger, Senegal, Uganda-CHEWs, Zambia-CHAs
**Supervision**	**Supervision by health facility workers/health post nurses**	**Supervision by other**
	Burkina Faso, Cameroon, DRC, Ethiopia, Ghana, Madagascar, Malawi, Mali, Mozambique, Niger, Rwanda, Senegal, Uganda, Zambia, Nicaragua,	Pakistan (lady supervisors attached to the health facility) DRC (site management committee, health zone staff, Malawi (senior health assistants), Ghana (zonal coordinators), Mali (local health committees), Nigeria (community health extension workers), Nepal (village health workers), Rwanda (cell coordinators), South Sudan (community recruited supervisors).

### Motivation of CHWs

A number of countries motivate their CHWs through financial incentives in the form of salaries and stipends (e.g. Ethiopia, Malawi, Mozambique, Pakistan). Several countries were in the process of pushing for the payment of CHWs (e.g. Burkina Faso, Niger, Uganda and Zambia). However, the sustainability of salaried CHW programs was questionable in some countries due to reasons discussed under financing above. For example, Niger was pushing for the inclusion of CHW stipends in its budget with a plan to finance 50% of the stipend, leaving the other 50% to the donors. However, this plan was rejected by some donor schemes, as salary and incentive payments are presumed to be the primary responsibility of the host country [30]. Some countries have both paid and unpaid CHWs (e.g. Zambia and Ghana). In such countries, the CHWs who provide iCCM tend to be paid. This has been reported to demotivate unpaid cadres of CHWs in some of the countries. In a limited number of countries, CHWs were allowed to sell drugs at a fee using full cost recovery models (e.g. Senegal) or partial cost recovery models (e.g. Burkina Faso previously, Madagascar). Village Health Committees (VHCs) manage the money generated from the drug sales in Senegal, with some of the money raised given to the CHWs as a bonus. CHWs in DRC, Ghana and Mali are allowed to charge small consultation fees which is given to the CHWs as salary in lieu.

Rwanda and Madagascar have community performance-based financing schemes (CPBF). The scheme in Madagascar is supported by the World Bank and is only implemented for a subset of CHWs who also raise awareness for maternal and neonatal health interventions [31,32]. The Rwanda MoH, with support from Global Fund, introduced the CPBF scheme in 2009 as a way to motivate CHWs and to improve quality and utilization of health services. Through the scheme, CHW cooperatives receive and share funds from the MoH every quarter based on their achievement of specific MoH-defined targets. Nearly three quarters of CPBF grants are invested into income generating activities for the cooperatives while 30% is shared out among CHWs as cash bonuses equivalent to approximately US$0.73 [22,24]. From a sustainability perspective, the Rwanda MoH contracted a local organization (Square Entrepreneurship Development Consult) to develop the business planning and financial management capacities of cooperatives, in preparation for the phasing out of the grants for performance-based financing from the Global Fund over time [22].

Other than salaries and stipends, there are no well-defined non-financial incentives described for CHWs in most country policy documents and implementation guidelines. When non-financial incentives are defined, they are often limited to ensuring availability of medicines and supplies; ensuring adequate supervision and recognition of CHWs; providing free health care; and low-cost incentives such as T-shirts. Although there are several studies that have been conducted on non-financial approaches to motivation of CHWs in African settings within specific implementation partner programs, there are no definitive standard models institutionalized by country governments. Also, non-financial incentives tend to vary from community to community. Table 2 summarizes the approaches used to motivate CHWs delivering iCCM.

### Supervision and performance quality assurance

Five approaches to supervision were described in the literature. One approach involves health facility staff supervising CHWs and this is used by almost all iCCM programs, either implemented on its own or together with other approaches. It was often aimed at checking on performance, ensuring quality, providing refresher training, report collection and replenishment of drugs. Another approach utilised iCCM project employees of implementing partners to supervise CHWs, e.g. Mali [33]. The third approach involved staff not based at the health facilities but attached to the health facility, such as zonal coordinators in DRC and Ghana, mainly for checking on performance, collection of iCCM reports and replenishment of drugs and supplies. The fourth mode of supervision comprised peer supervisors selected from among the CHWs and given extra supervisory duties and training, e.g. senior health assistants in Zambia, cell coordinators in Rwanda, community recruited supervisors in South Sudan, and lady health supervisors in Pakistan. This approach targeted report collection, and drug and supply replenishments. The last mode involved supportive supervision from village health committees, e.g. Malawi and Senegal [34]. Nearly all approaches to supervision involve either the supervisors visiting CHWs at their work post or at a health facility and vice versa, on an individual or group level. All approaches required some form of facilitation in terms of transport allowances. Table 2 further summarizes some of the approaches to supervision that were being used in the various countries.

### Supply chain management

Several approaches to procurement and supply management (PSM) for iCCM commodities were identified from the literature – see Table 3 for summary. First were the parallel iCCM PSM systems that were supported mainly by implementing partners. The systems were common to all countries in early stages of iCCM implementation and were developed to try and circumvent operational, logistical and infrastructure challenges experienced in the government-run systems. Second were the integrated PSM systems run by governments with support from their implementing partners and were implemented either through push or pull systems. In Ethiopia, the push system was mainly used to deliver iCCM medicines and supplies from drug stores to implementing sites in the early phases of the program implementation and was later switched to pull systems whereby drugs are drawn from central stores through requests of forecasted medicines based on consumption data [20]. Third were the predominantly government-run PSM systems exemplified by Nicaragua and Rwanda. Nicaragua was able to provide regular government financed iCCM medicines through its national PSM systems with the technical support of implementing partners even in the early implementation phases of its iCCM program [35]. In Rwanda, the Medical Procurement and Distribution Division of the MoH purchases medical supplies for the national health-care system. These medical supplies are stored in district pharmacy warehouses until distribution to district hospitals and health centers [24]. Lead CHWs known as cell coordinators operating at the lowest administrative unit, obtain the medicines from the health center and distribute them to CHWs in their catchment area based on consumption.

**Table 3.** Summary of iCCM procurement supply management models.

Government led iCCM PSM plan	Present	Absent	
	Burkina Faso, Ethiopia, Ghana, Madagascar, Malawi, Mozambique, Rwanda, Senegal, Uganda, Zambia, Nicaragua, Nepal, Pakistan	Cameroon, DRC, Mali, Nigeria, Niger, South Sudan	
**iCCM drugs supplied at the community level**	All countries supply iCCM drugs. In Burkina Faso drugs for Malaria are supplied at the community level and inclusion of drugs for pneumonia is underway.		
**System used**	**Parallel partner run system**	**Integrated pull and push-pull mechanism**	**Integrated push mechanism**
	DRC, Cameroon, Mali, Niger, Nigeria, South Sudan	Ethiopia, Ghana (CHOs may push medicines to CBAs), Madagascar, Senegal, Uganda (higher levels may push to lower levels), Zambia, Nepal (pull-push), Pakistan	Malawi, Mozambique, Rwanda, Burkina Faso
**PSM improvement tools and approaches**	**Forecasting and quantification**	**ehealth interventions**	**providing large stocks to hard-to-reach areas**
	Ethiopia, Ghana	Malawi (cStock), Mozambique (APE App), Rwanda (m’Ubuzima programe), Zambia.	South Sudan

*The integrated systems in all countries are partially supported by implementing partners.

### Tools for improved procurement supply chain management

The MoH of Rwanda together with its implementing partners developed tools for improved forecasting of community level demand of medical supplies to strengthen the PSM systems. The tools consisted of improved versions of paper-based resupply calculators and improved communication approaches designed by quality improvement teams [24,36]. In Malawi, similar efforts led to countrywide adoption of cStock, a simple mHealth reporting and resupply system that led to improvements in communication between the Health Surveillance Assistants (HSAs) and their resupply points. The application is reported to improve visualization of iCCM drug stocks at the district and central levels of the MoH through alert systems which enable supply chain managers to respond on a timely basis [36]. The Mozambique MoH together with its implementing partners are scaling up a digital community health system, called upSCALE, that enables supervisors to monitor medicine stokes by providing real-time data visualization [37]. There are several studies on the role of mHealth interventions in PSM for iCCM in other countries. However, the approaches recommended by these studies have not yet been institutionalized in national iCCM implementation on a large-scale [38].

### Service delivery and referral

#### Service packages

The strategies for iCCM program implementation in all the countries reviewed had core components required by the WHO/UNICEF joint statement on iCCM including artemisinin-based combination therapies (ACTs) and rapid diagnostic tests for malaria, oral antibiotics for pneumonia, low osmolality oral rehydration solution (ORS) and zinc tablets for diarrhea, referral of severe cases and the use of packaging designed for community-level use. However, other add-on components such as screening for acute malnutrition, referral of newborns and maternal follow-ups varied between countries, reflecting the national community health policies of the specific countries. The literature showed that in most countries there were frequent stock-outs of iCCM essential medicines and commodities affecting service delivery [36]. There was also evidence that referral linkages to health facilities existed in theory but in practice, several iCCM programs fell short in adherence to referral advice [39].

#### Provision of curative and promotive services

The modes of approach to preventive and curative services by CHWs varied from country to country. Some countries had specific policies reinforcing provision of health promotion activities by CHWs to avoid their abandonment due to the provision of curative services. Such policies were either based on time allocation basis or on redistribution of responsibility. For example, in Mozambique, Agentes Polivalentes Elementares (APEs) are obliged to spend 80% of their paid time on community health promotion activities and 20% on curative services [40]. Ethiopia’s HEW policy required that HEWs should spend at least 75% of their time at the health post and 25% in the community but this was changed to having at least one HEW at the health post during working hours after the establishment of the Health Development Army (HDA). The HDA of Ethiopia spend all of their time in the community conducting health promotion and disease prevention activities [20]. Malawi also has a special cadre of CHWs focusing on health promotion and disease prevention and Zambia is shifting towards this approach [20,25].

#### Places of service delivery

Broadly, there are two settings for service delivery described in the literature from which CHWs provide iCCM. The first setting consists of CHWs delivering iCCM in the communities while based at their homes (e.g. Burkina Faso, Cameroon, DRC, Nigeria, Rwanda, Uganda, and Pakistan (where they are called health houses)). The second approach involves CHWs based at a structure within the community (e.g. Ethiopia, Mali and Niger’s Health Posts, Ghana’s Community Health Compounds, Malawi’s Village Health Clinics and Senegal’s Health Huts). The settings in which CHWs provide iCCM are summarized under the characteristics of CHWs in Table 2.

#### iCCM service targets

Although iCCM was first targeted at primarily rural areas, some countries implement iCCM in a more targeted way on the basis of pre-designated hard-to-reach areas. For example, the Malawi MoH mapped out hard-to-reach areas located more than 8 kilometers away from a health facility which are supposed to be prioritized for implementation of iCCM by implementing partners [25]. Nicaragua used travel distance to the health facilities to prioritize the so-called type C communities that were located more than one hour from the health facility [35]. A summary of iCCM service delivery models is presented in Table 4.

**Table 4.** Service delivery models for iCCM and stage of implementation.

Stage of implementation	Early	Expansion	
	Cameroon, South Sudan	Burkina Faso, DRC, Ethiopia, Ghana, Madagascar, Malawi, Mali, Mozambique, Niger, Nigeria, Rwanda, Senegal, Uganda, Zambia, Nicaragua, Nepal, Pakistan	
**Health promotion responsibilities**	**Allocated to separate cadre of CHWs**	**Not allocated to separate cadre of CHWs**	**Mandatory partitioning of time**
	Cameroon, DRC, Ethiopia-HDAs, Zambia (only after obtaining a critical mass of CHAs), Niger (only in some areas), and Senegal	Burkina Faso, Ghana, Madagascar, Malawi, Mali, Nigeria, Rwanda, South Sudan, Uganda, Nicaragua, Nepal and Pakistan.	Ethiopia-mandatory time partitioning, Mozambique- 80% CHW work must be on health promotion
**Provision of curative services**	**Free services for malaria, pneumonia and diarrhea**	**Free services for malaria, pneumonia and diarrhea but with user charges**	**Paid for services**
	Burkina Faso (recently transitioned from paid services), Cameroon, Ethiopia, Malawi, Mali (only for pneumonia), Mozambique, Niger, Nigeria, Rwanda, South Sudan, Uganda, Zambia, Nicaragua, Nepal and Pakistan	DRC, Ghana, Mali, and Senegal	Madagascar (subsidised Medicine charges), Mali (diarrhea and pneumonia)

#### Monitoring, evaluation and health information systems

Most countries rely on national health management information management systems (HMIS) to monitor the delivery and utilization of services at health facilities and the community level. However, functional community health information systems require a proper monitoring and evaluation plan [41,42]. The documents reviewed showed that some countries had full iCCM M&E plans (e.g. Ethiopia, Ghana, Malawi, Niger, Nigeria, Rwanda, and Uganda) while others had partial or no M&E plans (e.g. Mali, Mozambique, DRC, Zambia and South Sudan). With respect to HMIS, iCCM indicators were already integrated into the national HMIS either fully (e.g. Niger, Nigeria, Rwanda, Uganda), partially (e.g. Mozambique), or not at all (e.g. Zambia, South Sudan). For some countries, iCCM data were already being reported in the national HMIS and could be disaggregated by community level. This disaggregation is for all iCCM conditions in some countries (e.g. Ghana, Malawi, Niger, Nigeria, Rwanda and Uganda), for some of the conditions in others (e.g. Ethiopia and DRC), or not at all (including Mali, Mozambique, Zambia and South Sudan). In some countries, iCCM data were entered into a parallel dataset at the subnational level for future integration into the HMIS at the national level. In other countries, iCCM data were entered into the HMIS system at the district level via the DHIS2 (e.g. Uganda, Nigeria and DRC). In most countries, iCCM data from the HMIS could not be traced to individual CHWs. The data were also usually incomplete due to operational, logistical and infrastructure challenges [41–43].

Remarkably, there were promising simple paper and technology-based tools that had been developed to improve the quality of community data. For example, the use of the Lot Quality Assurance Sampling (LQAS) tool in Rwanda and other countries created an opportunity for data quality improvement [44]. Similarly, the use of mobile-based technologies in national community-level data collection (such as mTrac in Uganda, cStock in Malawi and upSCALE in Mozambique) provided opportunities for improved data quality by limiting human errors [37,41,45].

#### Communications and social mobilization

In most countries, communication and mobilization for increased iCCM demand was through community engagement. It often involved health promotion messages delivered by CHWs who provide iCCM to community members through home visits. Religious and political institutions were also mentioned in some countries as purveyors of iCCM messages, e.g. in Cameroon, DRC, Niger and Mozambique [46,47]. Several countries have a special cadre of CHWs dedicated to providing health promotion and behavior change communication (BCC) messages, e.g. relais communautaires of Senegal, DRC, Cameroon, Mali, Niger [47,48]. In DRC, church volunteers carry out BCC in addition to the relais communautaires [46]. In Malawi and Senegal, the VHCs encourage people to participate in health-related projects [34].

## Discussion

iCCM has been shown through implementation and programmatic experience to be a key public health strategy as a cost-effective approach for achieving high coverage of quality treatment services for young children. There have some previous assessments and reviews of iCCM which have been done from a health systems perspective, and have stressed the importance of appropriate policies and system-wide approaches for sustainable and contextualized programs [49,50].

This review provides a synthesis of the approaches that have been used to institutionalize iCCM into national health systems from both available grey and published literature. An overview of the lessons learned from the successes and limitations of the various approaches that have been used by government departments to institutionalize benchmark components of iCCM into national health systems has been generated. A summary of the compliance by countries with these benchmark components is presented in Table 5 where examples of best practice are highlighted against each component. Subsequently, recommendations for future program sustainability have been made, which are additionally informed by the authors’ experience of implementing iCCM and interactions with collaborating partners, including MoHs.

**Table 5.** Compliance with iCCM benchmark components by low to middle-income countries.

	Benchmark components of integrated community case (iCCM)							
*Countries	Coordination and policy setting	Costing and financing;	Human resources (CHWs)	Supply chain management	Service delivery and referral	Communications and social mobilization	Supervision and performance quality assurance	Monitoring, evaluation and integration into health information systems
**East Africa**								
Ethiopia	☑	☑	☑	☑	✓	✓	✓	☑
Madagascar	✓		✓	☑	✓		✓	
Malawi	☑	☑	☑	☑	✓	☑	✓	☑
Mozambique			☑	☑	✓	✓	✓	✓
Rwanda	☑	☑	☑	☑	✓	✓	✓	☑
Uganda	✓		☑	☑	✓	✓	✓	☑
Zambia	✓	☑	☑	☑	✓	✓	✓	✓
South Sudan		☑	✓	✓	✓	✓	✓	✓
**Central Africa**								
Cameroon	✓	☑	✓	✓	✓	☑	✓	
DRC	✓	☑	✓	✓	✓	☑	✓	✓
**West Africa**								
Burkina Faso	✓		☑	☑	✓	☑	✓	
Ghana	☑	☑	☑	☑	✓	✓	✓	☑
Mali	✓		✓	✓	✓	☑	✓	✓
Niger	✓		✓	✓	✓	☑	✓	☑
Nigeria	✓	☑	✓	✓	✓		✓	☑
Senegal	✓	☑	✓		✓	☑		
**Asia**								
Nepal	✓		☑	☑	✓		✓	✓
Pakistan			☑	✓	✓		✓	✓
**Central America**								
Nicaragua	✓		✓	☑	✓		✓	✓

☑Best practice examples, ✓Otherwise

### Lessons learned and recommendations

#### Policy and governance

While the existence of separate iCCM policies or policies spread across various government departments is a step in the right direction, sustainable implementation of iCCM requires strong government ownership at both local and central government levels. Policies and strategies for iCCM implementation housed in different government ministries and departments need to be well streamlined. It is necessary to have an appropriate department within government or ministry structures that houses iCCM. Creation of parallel external partner-dependent iCCM programs that try to circumvent the difficulties encountered by the national health system at the expense of health system strengthening should be discouraged. Failure to allocate an appropriate department for iCCM is likely to generate lack of harmonization among departments that work on the various aspects of iCCM, such as child health, malaria control and primary health programs. Whilst partner-run programs may be necessary in the early and interim phases of iCCM implantation, there needs to be unified central policies that guide implementation by the many partners [11]. iCCM ought to be embedded in national community-based primary health-care strategies with strong management and governance accountability.

#### Financing of iCCM

Government-led financing of iCCM is crucial for sustainability although donor funding is usually necessary in early and interim phases of iCCM implementation. Several iCCM programs are heavily reliant on donors and implementing partners for their survival. However, donors are increasingly demanding more commitment from governments to fund recurrent iCCM costs. The need for increased advocacy for domestic funding right from the outset of iCCM programs cannot be overstated.

Government commitment to financing should be evident through inclusion of iCCM in national health budgets, with well-costed plans and scale-up roadmaps outlining affordable investments that should be made. It is known that iCCM programs only make economic sense when adequately utilized. Government-led programs are therefore expected to expand opportunities for access to quality-assured services that generate demand for iCCM within the private and public sector [26].

There is evidence that community health insurance schemes improve domestic health financing and communities play an important role in financing of iCCM through offering support to CHWs and in-kind payments. However, community-led financing initiatives for iCCM are generally lacking and ought to be encouraged.

#### Supervision and workload of CHWs

Supervision is essential for performance, motivation and quality assurance. Most models rely on already overburdened health facility-based staff for all types of supervision, who take on supervision roles in addition to their clinical and administrative roles. Several countries are moving toward iCCM implementation models whereby CHWs providing curative services are typically of a higher cadre, receiving either a salary or some form of financial incentives, and have a general responsibility to supervise lower cadres of CHWs who may be salaried or not. The creation of such cadres of iCCM CHWs serves to relieve health facility staff from some of their supervisory responsibilities hence improving efficiency in reporting and replenishment of medicines and supplies. Still, there is a need to establish an iCCM supervision structure with a designated individual responsible for the technical supervision and coordination of CHWs, e.g. Health Assistants in Uganda. Moreover, such models do not completely eliminate some of the key challenges with supervision often mentioned in the literature, such as lack of transport for supervision visits [34,51].

Some countries have moved towards use of community-based models for supervision such as the village health committee models in Senegal and Mali. Apart from this, alternative methods for supervision of CHWs that can potentially lead to increased community ownership such as community monitoring, are rarely stipulated in national supervision strategies. While CHWs need several forms of supervision, national policy strategies tend to be focused on only technical supervision. Expanding supervision strategies to include community-based structures can improve iCCM supervision and the overall community ownership of iCCM [34,51].

Several countries are also moving towards models where health promotion and preventive services are partially separated by the cadre of CHWs who deliver them. The partial separation of curative and preventive services will help lessen workload for CHWs delivering curative services such as iCCM. Since both curative and preventive services are necessary for successful scale-up of iCCM programs and community-based primary health care in general, such models could strengthen promotive and preventive services.

### Motivation of CHWs

Motivation of CHWs is important for retention. However, many countries are struggling with the maintenance of financial incentives for CHWs. Innovative ways of financing CHW incentives such as the government-led community performance-based financing scheme in Rwanda and other micro-finance schemes need to be explored as viable remuneration approaches [52]. The alternative to financial incentives is non-financial motivation but many countries without financial incentives have no well-defined motivation packages. Furthermore, alternative approaches to motivation such as those based on non-financial motivation and intrinsic motivators of CHWs need to be taken into consideration when designing iCCM programs.

### Service delivery and referral

Approaches to delivery of iCCM services by CHWs should be based on the institutionalization of iCCM into community-based health service delivery as an embedded strategy to extend and complement facility-based health service delivery. This should form a coherent community-based primary health-care strategy. This will lead to improved linkages between health facilities and community structures, enhancing referral completion and smooth flow of essential medicines and commodities for iCCM [5].

iCCM implementation could be more targeted rather than universal, so that the services reach just those truly without access. Better targeting provides opportunities for improved allocative efficiency, improved outcomes and more affordable iCCM programs [35].

### Procurement and supply management systems

Uninterrupted PSM models are essential for effective delivery of iCCM. As much as procurement systems that are parallel to the national system seem efficient in the short term, there is a need to always procure medicines through the existing government systems since parallel systems are contradictory to health system strengthening principles and not sustainable in the long run. It will be important to invest in promising mHealth technologies and social innovations that have proved to improve PSM. Some social innovations include Nicaragua’s targeted prioritization of iCCM for hard-to–reach areas using government medicine stocks and Rwanda’s simple form revisions that led to better forecasting of medicine stocks. Exemplary mHealth innovations include Malawi’s cStock and Mozambique’s upSCALE innovations [35–37].

### Health information systems

Routine monitoring of health programs is important to track progress and identify issues in implementation. Development of comprehensive monitoring and evaluation plans that track all priority routine indicators at the various levels of the health system is essential. These ought to be complemented by appropriate improvements in tools capturing relevant community-level indicators, which are useful for both operational and programmatic decision-making. Investment in technologies that minimize structural, infrastructural and human errors in the computation and reporting of community health information system indicators is necessary. It is also essential to ensure that data can be disaggregated and used to give feedback at various levels of the health system [41].

Finally, country-specific iCCM implementation research drawing on various sources including national community data is still needed to provide answers about the quality of iCCM care and its cost-effectiveness, as well as its long-term positioning within the primary healthcare strategy.

### Study limitations

The inclusion criteria eliminated documents written in languages other than English. Nonetheless, it should be noted that some of the documents available in other languages were sometimes referenced in the English literature and therefore the reviewers captured some of the approaches reported in the articles. Scoping reviews are inherently limited by their inability to appraise the methods of studies included. However, this study only sought to provide an overview of the approaches that have been used by countries to institutionalize iCCM into their national health systems. Scoping reviews can be enriched by an optional consultative exercise with stakeholders to validate results. Although this exercise was omitted, interpretation of results was informed by the authors’ long experience implementing iCCM and interactions with collaborating partners, including MoHs.

## Conclusion

Although iCCM policies exist in most countries, and various methods have been applied, it often remains poorly institutionalized into national health systems in different contexts. Heavy donor funding and implementation partner dependence characterize iCCM programs. Implementation methods of iCCM benchmark components employed by partners are sometimes parallel to national health systems and conflicting with health system strengthening principles. Improved government ownership of iCCM programs at central and sub-national levels is required to ensure uninterruptible supplies of quality-assured commodities for iCCM. This should be complemented by beneficiary community participation and ownership.

## Paper context

iCCM is a recommended strategy for addressing child mortality. To date, most iCCM programs are run by international and local non-governmental organizations that operate on a small scale. Even in countries where the programs are implemented to a large scale, the programs are still heavily dependent on funding from external donors. This scoping review draws on lessons learned from government-led programs to provide recommendations for large-scale institutionalization of iCCM into national health systems.
